# Astaxanthin Counteracts Excitotoxicity and Reduces the Ensuing Increases in Calcium Levels and Mitochondrial Reactive Oxygen Species Generation

**DOI:** 10.3390/md18060335

**Published:** 2020-06-26

**Authors:** Francisca García, Pedro Lobos, Alejandra Ponce, Karla Cataldo, Daniela Meza, Patricio Farías, Carolina Estay, Felipe Oyarzun-Ampuero, Rodrigo Herrera-Molina, Andrea Paula-Lima, Álvaro O. Ardiles, Cecilia Hidalgo, Tatiana Adasme, Pablo Muñoz

**Affiliations:** 1 Laboratory of Cellular and Molecular Plasticity, Department of Pathology and Physiology, Medical School, Faculty of Medicine, Universidad de Valparaíso, Valparaíso 2341386, Chile; fjgr14@gmail.com (F.G.); alejandra.poncecid@gmail.com (A.P.); karlacataldo@gmail.com (K.C.); danita.meza@gmail.com (D.M.); pato.fras@gmail.com (P.F.); carito.estay@gmail.com (C.E.); alvaro.ardiles@uv.cl (Á.O.A.); 2 Translational Neurology Center, Faculty of Medicine, Universidad de Valparaíso, Valparaíso 2341386, Chile; 3 Biomedical Research Center, Universidad de Valparaíso, Valparaíso 2341386, Chile; 4 Biomedical Neuroscience Institute, Faculty of Medicine, Universidad de Chile, Santiago 8380000, Chile; ploboszqf@gmail.com (P.L.); acpaulalima@u.uchile.cl (A.P.-L.); chidalgo@med.uchile.cl (C.H.); 5 Department of Technology and Pharmaceutical Sciences, Faculty of Chemical and Pharmaceutical Sciences, Advanced Center for Chronic Diseases (ACCDiS), Universidad de Chile, Santos Dumont 964, Independencia, Santiago 8380494, Chile; foyarzuna@ciq.uchile.cl; 6 Leibniz Institute for Neurobiology, 39118 Magdeburg, Germany; rherrera@lin-magdeburg.de; 7 Centro Integrativo de Biología y Química Aplicada (CIBQA), Universidad Bernardo O’Higgins, Santiago 8370854, Chile; 8 Institute for Research in Dental Sciences, Faculty of Dentistry, Universidad de Chile, Santiago 8380000, Chile; 9 Interdisciplinary Center of Neuroscience of Valparaíso, Universidad de Valparaíso, Valparaíso 2381850, Chile; 10 Interdisciplinary Center for Health Studies, Universidad de Valparaíso, Valparaíso 2341386, Chile; 11 Department of Neurosciences and Program of Physiology and Biophysics, Institute of Biomedical Sciences, Faculty of Medicine, Universidad de Chile, Santiago 8380000, Chile; 12 Center for Exercise, Metabolism and Cancer Studies, Faculty of Medicine, Universidad de Chile, Santiago 8380000, Chile

**Keywords:** NMDA, astaxanthin, calcium, mitochondrial superoxide, excitotoxicity

## Abstract

Astaxanthin (ASX) is a carotenoid pigment with strong antioxidant properties. We have reported previously that ASX protects neurons from the noxious effects of amyloid-β peptide oligomers, which promote excessive mitochondrial reactive oxygen species (mROS) production and induce a sustained increase in cytoplasmic Ca^2+^ concentration. These properties make ASX a promising therapeutic agent against pathological conditions that entail oxidative and Ca^2+^ dysregulation. Here, we studied whether ASX protects neurons from N-methyl-D-aspartate (NMDA)-induced excitotoxicity, a noxious process which decreases cellular viability, alters gene expression and promotes excessive mROS production. Incubation of the neuronal cell line SH-SY5Y with NMDA decreased cellular viability and increased mitochondrial superoxide production; pre-incubation with ASX prevented these effects. Additionally, incubation of SH-SY5Y cells with ASX effectively reduced the basal mROS production and prevented hydrogen peroxide-induced cell death. In primary hippocampal neurons, transfected with a genetically encoded cytoplasmic Ca^2+^ sensor, ASX also prevented the increase in intracellular Ca^2+^ concentration induced by NMDA. We suggest that, by preventing the noxious mROS and Ca^2+^ increases that occur under excitotoxic conditions, ASX could be useful as a therapeutic agent in neurodegenerative pathologies that involve alterations in Ca^2+^ homeostasis and ROS generation.

## 1. Introduction

Astaxanthin (ASX) is an orange-red carotenoid pigment produced by the microalgae Haematococcus pluvialis, the red yeast Phaffia rhodozyma and other marine species [1]. Due to its strong anti-oxidant properties, ASX has been considered as a promising molecule for the treatment of inflammation, cancer, and age-related diseases [2,3,4]. A remarkable property of ASX is that, unlike many other natural antioxidants, it readily crosses the blood–brain barrier; due to this property, ASX has been used successfully in rodents to reverse both ischemia-reperfusion-induced brain damage [5] and cognitive impairment [6,7]. In neuron-like HT22 cells [7], PC12 cells [8] or SH-SY5Y cells [9,10,11] ASX suppresses the excitotoxic responses induced by glutamate, 6-hydroxydopamine (6-OHDA), 1-methyl-4-phenyl-1, 2, 3, 6-tetrahydropyridine (MPTP) or acetaldehyde. Although the neuroprotective effects of ASX might involve decreasing oxidative stress and improving mitochondrial integrity [12], whether ASX induces adaptive responses such as changes in redox-sensitive cellular signaling processes-including calcium (Ca^2+^)-dependent signaling pathways and reactive oxygen species (ROS) generation remains largely unknown.

Glutamate is the most abundant neurotransmitter of the mammalian central nervous system. This amino acid participates in excitatory neurotransmission processes by activating ionotropic or metabotropic glutamate receptors [13]. In particular, the N-methyl-D-aspartate (NMDA) glutamate receptor (NMDAR) is a subtype of ionotropic glutamate receptor that contributes to neuronal processes such as synaptic plasticity, which converts neural activity patterns into long-term changes in the structure and function of synapses [14]. In this context, Ca^2+^ influx through NMDAR is amplified and propagated by a mechanism known as Ca^2+^-induced Ca^2+^ release (CICR), a process in which the intracellular Ca^2+^ channels resident in the endoplasmic reticulum, Ryanodine Receptors (RyR) and Inositol 1,4,5-trisphosphate receptors (IP_3_R), have a central role [15]. Activation of these Ca^2+^ channels triggers different Ca^2+^-dependent processes, including the activation of key transcription factors, changes in gene expression, structural changes in dendritic spines and memory formation and loss [16,17]. In particular, RyR-mediated Ca^2+^-release participates in the acquisition and/or consolidation of spatial memory processes [17]. Interestingly, the physiological activation of NMDAR, which has been associated with moderate increments of neuronal ROS levels [18], promotes cell survival [19] by similar mechanisms to those involved in the establishment of synaptic plasticity [20,21].

On the other hand, excessive release or chronic increases in glutamate levels generate NMDAR-mediated excitotoxicity, a non-physiological response which occurs in stroke [22] and neurodegenerative conditions such as Alzheimer’s, Parkinson’s and Huntington diseases [23]. NMDAR over-activation, as observed in excitotoxic conditions, leads to the deregulation of Ca^2+^ homeostasis [24,25]. Excessive intracellular Ca^2+^ levels, in turn, lead to mitochondrial membrane potential loss, opening of the mitochondrial transition pore, ROS overproduction, inhibition of respiratory chain enzymes and the consequent decrease in ATP synthesis [26]. As a result of this oxidative state imbalance, the mitochondria are unable to re-establish the transmembrane ion gradients and thus generate ATP, causing cell death [27].

Several reports indicate that redox-sensitive RyR-mediated CICR plays a key role in synaptic plasticity, including structural plasticity [28], as well as in the impairment of processes involving the deregulation of intracellular Ca^2+^ concentration ([Ca^2+^]) [29]. Thus, CICR plays a key role in hippocampal neuronal function, both at the physiological level as part of the mechanisms underlying structural and synaptic plasticity [28], and as part of the imbalance of Ca^2+^ and ROS levels that occurs during aging [30] or in pathological conditions such as Alzheimer’s disease [31].

Currently, intensive research efforts are dedicated to find compounds with biological activity against oxidative stress and excitotoxicity in neurons. In this work, we studied the effects of ASX in NMDAR-mediated excitotoxicity. To this aim, we evaluated the effects of ASX on mitochondrial ROS production, cell viability and intracellular Ca^2+^ levels in cells treated with excitotoxic NMDA concentrations. We found that ASX decreased mitochondrial ROS levels and alleviated the mitochondrial dysfunction induced in SH-SY5Y cells by NMDA and also prevented H_2_O_2_-induced cell death. In addition, ASX attenuated the [Ca^2+^] increase displayed by primary rat hippocampal neurons treated with NMDA.

## 2. Results

### 2.1. Dose-Dependent Activation of NMDAR Increases Cytoplasmic [Ca^2+^] and Causes Excitoxicity in SH-SY5Y Cells

SH-SY5Y cells, a human neuroblastoma cell line derived from the catecholaminergic line SK-N-SH [32], have been widely used as an in vitro model for Parkinson’s disease and other neuronal disorders. Although some studies suggest the complete absence (or lower expression) of NMDAR in SH-SY5Y cells [33], other reports have shown that these cells express both ionotropic and metabotropic glutamate receptors [34]. Moreover, SH-SY5Y cells expressing glutamate receptors have been used for studying glutamate- and NMDA-mediated excitotoxicity [35].

**Figure 1:**
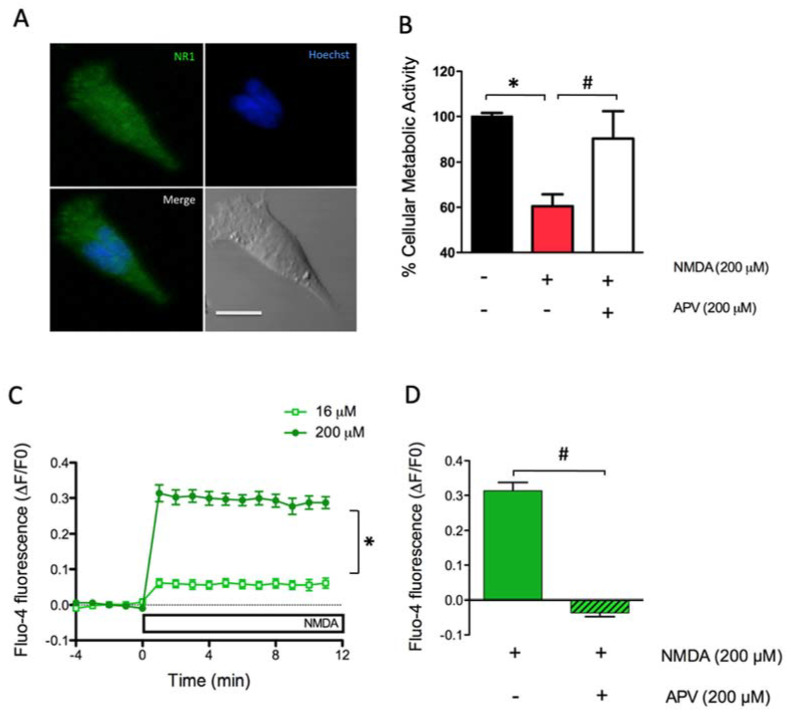
Cell viability and Ca^2+^ levels induced by N-methyl-D aspartate (NMDA) addition to SH-SY5Y cells. (**A**) Representative immunofluorescence images showing the presence of the NMDA glutamate receptor (NMDAR) subunit GluR1/N1. Cells were fixed, permeabilized and incubated with a specific antibody against the GluR1/N1 subunit of the NMDAR (green). The nucleus was stained with Hoescht (blue). The phase-contrast image at the lower right presents the 10 µM calibration bar. (**B**) Quantification of cell viability evaluated 24 h after treatment for 120 min with 200 µM NMDA. Additionally, cells were pre-incubated for 1 h with 200 µM D-2-Amino-5-phosphonovaleric acid (APV), an NMDA receptor antagonist. The graph shows the % cell viability. The black, red and white bars correspond to unstimulated cells, to cells stimulated with NMDA or to cells stimulated with NMDA in the presence of APV, respectively; the * indicates significant difference (*p* < 0.05) compared to control: ^#^ compared to 200 µM NMDA plus 200 µMAPV. (**C**) NMDA-induced Ca^2+^ signals in SH-SY5Y cells loaded with Fluo-4 and treated with 16 µM or 200 µM NMDA. Data were normalized against the fluorescence values obtained before NMDA addition (∆F/F0); *: *p* < 0.05 compared to 16 µM NMDA. (**D**) Ca^2+^ levels detected one minute after addition of 200 µM NMDA, or after addition of 200 µM NMDA to cells pre-incubated for 1 h with 200 µM APV; ^#^: *p* < 0.05 compared to NMDA-treated cells.

To detect the presence of NMDAR in undifferentiated SH-SY5Y cells, we performed an immunofluorescence analysis using a specific antibody against the NR1 subunit of the NMDAR. A representative image, illustrated in Figure 1A, shows that SH-SY5Y cells exhibit significant NR1 levels, detected as a green fluorescent label, indicating that our cell SH-SY5Y line expresses NMDAR, as previously reported in other studies [33,34,35]. A negative control lacking the primary antibody allowed us to exclude the possibility of non-specific NMDA staining (Supplementary Figure S3).

Cellular metabolic activity was evaluated after incubation of SH-SY5Y cells with tetrazolium (MTT) salt, which becomes reduced in cells with metabolically active mitochondria. Although the reduction process is not exclusively mitochondrial; it occurs necessarily in living cells, so the MTT assay has been widely used as a marker of cell viability [36]. MTT reduction was evaluated 24 h after treatment with NMDA for 2 h. We confirmed the harmful effects of the excitotoxic conditions caused by incubating SH-SY5Y cells with 200 µM NMDA for 2 h, since in these conditions cell metabolic activity decreased to 60.5 ± 5.3% of control (Figure 1B). Pre-incubation for 1 h with 200 µM APV prevented the decrease in cell metabolic activity induced by NMDA, which reached values of 90.4 ± 11.9% relative to the controls. The protective effects of APV indicate that NMDAR mediate the cell metabolic activity impairments induced by treatment for 2 h with 200 µM NMDA (Figure 1B).

Activation of NMDAR in the primary hippocampal neurons generates a measurable increase in cytoplasmic [Ca^2+^] [21]. Hence, we next evaluated the cytoplasmic [Ca^2+^] levels after NMDA addition to SH-SY5Y cells previously loaded with the Ca^2+^ probe Fluo 4-AM. Figure 1C shows that stimulation with a low concentration of NMDA (16 µM) produced a discrete increase in Fluo-4 fluorescence levels (0.061 ± 0.014), while stimulation with 200 µM NMDA produced a robust increase in Fluo-4 fluorescence (0.287 ± 0.017). Quantification of fluorescence intensity for each condition was expressed as the average fluorescence intensities, obtained after one minute of stimulation with 200 µM NMDA. Preincubation with 200 µM APV prior to the addition of 200 µM NMDA (Figure 1D), fully prevented the [Ca^2+^] increase induced by NMDA (NMDA = 0.314 ± 0.024 v/s NMDA + APV = −0.037 ± 0.011), an indication that NMDAR activation mediates the intracellular [Ca^2+^] elevation induced by NMDA.

### 2.2. Long-Term Treatment with ASX Protects SH-SY5Y Cells Against Neurotoxic Stimuli

**Figure 2:**
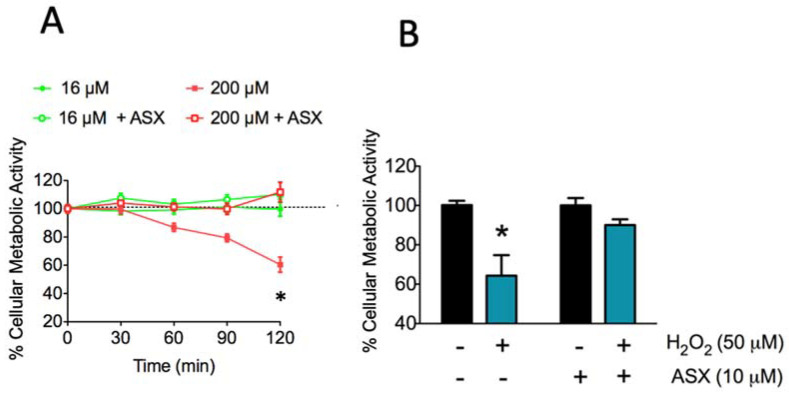
Effect of ASX on SH-SY5Y cell metabolic activity. (**A**) Cells were preincubated for 24 h with vehicle (closed symbols) or with 10 µM ASX (open symbols); following this period, cells were incubated with 16 µM NMDA (green symbols) or 200 µM NMDA (red symbols) for up to 2 h, as indicated in Figure 2A. Cellular metabolic activity was determined 24 h after incubation with NMDA using the MTT assay. ^#^: *p* < 0.05 when comparing 200 µM NMDA with 200 µM NMDA + ASX. (**B**) Cells were pretreated with vehicle or 10 µM ASX for 24 h; following this period 50 µM H_2_O_2_ was added for 1 h and cellular metabolic activity was measured 24 h later using the MTT assay. *: *p* < 0.05 compared to vehicle.

ASX has been used to improve mitochondrial integrity and combat oxidative stress [12] due to the fact that it has a higher antioxidant capacity than other carotenoids of the same family [37]. This ASX property resides in its chemical structure. Here, we investigated whether the treatment of SH-SY5Y cells with 10 µM ASX for 24 h before NMDA addition preserved cellular metabolic activity (Figure 2A, open symbols).

We found that incubation of SH-SY5Y for up to 2 h with a low concentration of NMDA (16 μM), closer to the physiological range, also increased both [Ca^2+^] and mitochondrial superoxide levels but did not affect cellular metabolic activity (Figure 2A, green solid circles); similarly, primary hippocampal neurons incubated for 2 h with 16 μM NMDA displayed the same metabolic activity as controls (Supplementary Figure S2). Likewise, cells preincubated for 24 h with ASX and then incubated with 16 μM NMDA displayed on average 100% metabolic activity (Figure 2A, green open circles). In contrast, cells treated with 200 μM NMDA (red solid squares) displayed a drastic decrease in metabolic activity, reaching values near 60% of control after 120 min incubation with NMDA. Preincubation of cells with 10 μM ASX for 24 h completely prevented the cell death induced by 200 µM NMDA (Figure 2A, red open squares). Consistent with its reported antioxidant effects, Figure 2B shows that preincubation with 10 μM ASX for 24 h fully prevented the decrease in cellular metabolic activity caused by 1 h incubation with 50 μM H_2_O_2_ (in %, H_2_O_2_: 64.3 ± 10.5; H_2_O_2_ + ASX: 91.2 ± 2.5). Of note, as illustrated in Figure 2B, incubation for 24 with 10 μM ASX did not induce significant changes in cellular metabolic activity with respect to control cells incubated with vehicle (in %, ASX: 100.0 ± 3.7; control: 100.0 ± 2.2).

### 2.3. Astaxanthin Decreases Mitochondrial ROS Levels in SH-SY5Y Cells

**Figure 3:**
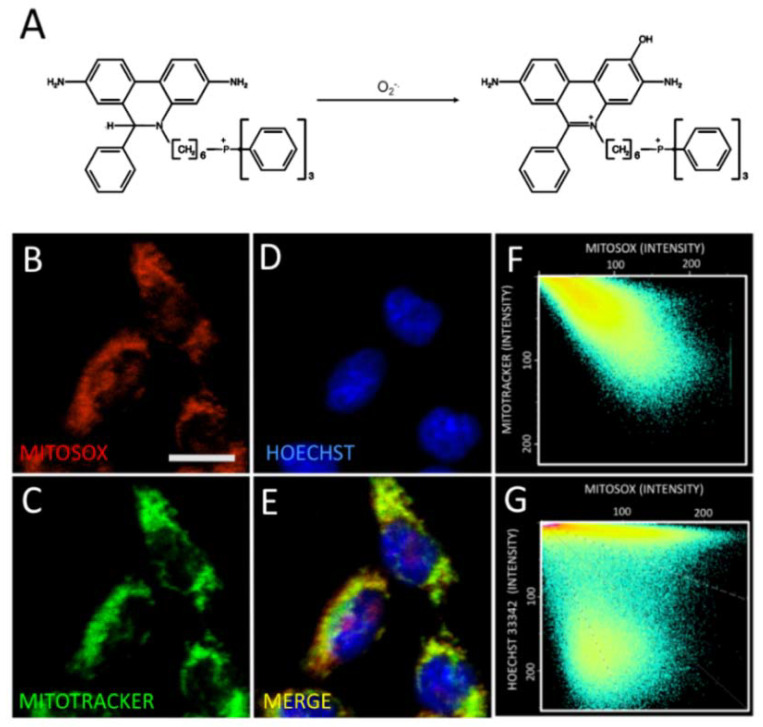
Mitochondrial Superoxide detection. (**A**) Reaction scheme of the mitochondrial superoxide sensor and its adduct. SH-SY5Y cells were simultaneously loaded with MitoSOX (**red color**, **B**), Mitotracker green (**green color**, **C**) and Hoescht (**blue color**, **D**). The composite image for MitoSOX, Mitotracker green and the Hoescht stain is shown in panel **E**. The pixel intensity analysis for the red v/s the green label and for the red v/s the blue label is shown in panels **F** and **G**, respectively.

To determine whether the incubation of SH-SY5Y cells with NMDA promotes mitochondrial ROS generation, we evaluated first the specificity of the superoxide-sensitive mitochondrial probe MitoSOX (Figure 3A,B, red label) by comparing its distribution with that of the known mitochondrial marker Mitotracker green (Figure 3C, green label). Image analysis to test for the colocalization of both probes showed that the mitochondrial marker Mitotracker green displayed a similar localization pattern as the MitoSOX probe (Figure 3F), confirming the specific mitochondrial localization of the MitoSOX probe. We also stained cells with the nuclear marker Hoechst (Figure 3D, blue) and compared the pattern displayed by the mitochondrial MitoSOX probe against the nuclear marker Hoechst (Figure 3G). Through this colocalization analysis, we corroborated that the MitoSOX probe targets the mitochondria in our SH-SY5Y cellular model.

**Figure 4:**
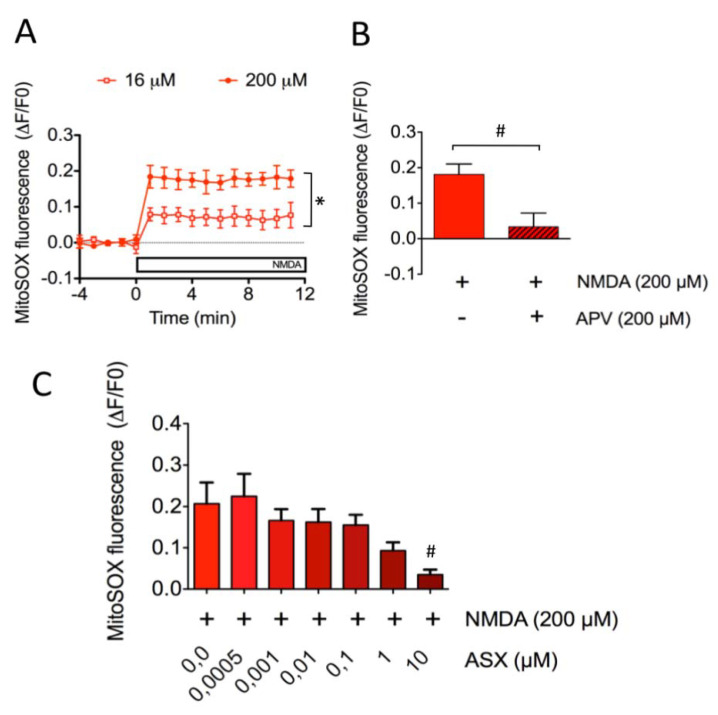
Protective effects of ASX on NMDA-induced mitochondrial superoxide generation in SH-SY5Y cells. (**A**) Mitochondrial superoxide levels were detected as a function of time with the MitoSOX probe following addition of 16 µM or 200 µM NMDA. Data were normalized against the fluorescence values obtained before NMDA addition (∆F/F0). *: *p* < 0.05 compared to addition of 16 µM NMDA. (**B**) Mitochondrial superoxide levels were measured one minute after addition of 200 µM NMDA to control cells or to cells preincubated for 60 min with 200 µM APV. ^#^: *p* < 0.05 compared to NMDA. (**C**) Cells pretreated with increasing concentrations of ASX (from 0.5 nM to 10 µM) for 24 h were incubated with 200 µM NMDA. ^#^: *p* < 0.05 compared to 200 µM NMDA in the absence of ASX.

Next, we tested whether incubation of SH-SY5Y cells with NMDA promoted mitochondrial ROS generation. To this aim, cells were loaded with the MitoSOX probe and mitochondrial superoxide levels were determined after NMDA addition. As illustrated in Figure 4A (open squares), SH-SY5Y cells incubated with 16 µM NMDA displayed a discrete elevation of mitochondrial ROS levels (0.077 ± 0.035), whereas cells treated with 200 µM NMDA (Figure 4A, closed circles) displayed a marked increase in these levels (0.179 ± 0.024). The mitochondrial ROS generation induced by 200 µM NMDA did not occur in cells incubated with APV (NMDA: 0.181 ± 0.029 v/s NMDA + APV: 0.034 ± 0.038) (Figure 4B), indicating that NMDAR activation mediated the ROS increase produced by NMDA.

Next, to determine if ASX prevented NMDA-induced mitochondrial ROS generation, we determined mitochondrial ROS levels in cells incubated with 200 µM NMDA and previously treated for 24 h with increasing ASX concentrations, from 0.5 nM to 10 μM. We found that cells preincubated with ASX displayed in a dose-dependent decrease in superoxide levels in response to NMDA addition (Figure 4C); cells preincubated, with 10 μM ASX displaying the lowest values (NMDA: 0.207 ± 0.051 v/s NMDA + ASX: 0.035 ± 0.012).

Based on these combined results, we suggest that the pre-incubation of cells with ASX for 24 h induces a metabolic response entailing adaptive changes in cellular redox state, which prevent or counteract the neurotoxic insult and the decreased cellular metabolic activity induced by excitotoxic concentrations of NMDA.

### 2.4. Astaxanthin Attenuates the Generation of Excitotoxic Ca^2+^ Signals in Primary Hippocampal Neurons

**Figure 5:**
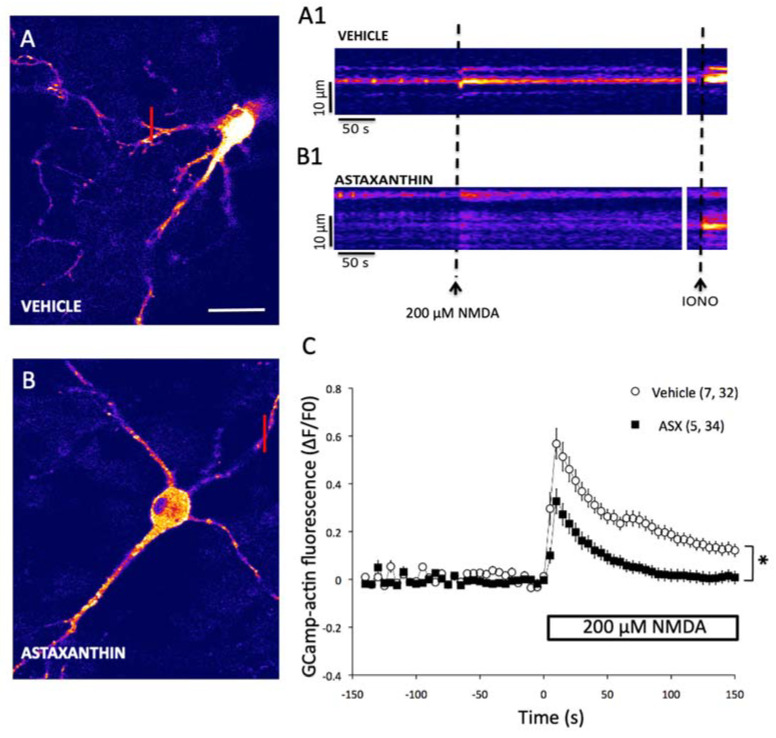
Effects of ASX on NMDA-induced calcium signal generation in primary hippocampal neurons. (**A**) Representative confocal image of a hippocampal neuron (7 DIV) transfected with the genetically encoded calcium sensor GCamP3-actin; the image was collected at the peak of the fluorescence increase induced by 200 µM NMDA. Calibration bar: 40 nm. (**B**) Representative confocal image of a neuron cultured in similar conditions as in A, except that the culture was pre-incubated for 24 h with 10 µM ASX prior to addition of 200 µM NMDA. Representative line scan images generated by collecting every 1-s from the dendritic region indicated by the red line drawn in the control image (**A1**) or in the image of ASX-pretreated neuron (**B1**); the addition of 200 µM NMDA and of 100 µM Ionomycin (IONO) is indicated by a dotted black line. (**C**). Data were normalized against the fluorescence values obtained before NMDA addition (∆F/F0). The graph shows the fluorescence intensity as a function of time collected from the neuronal control (*n* = 7; 32 ROIs) and from neuronal cells pretreated with ASX (*n* = 5; 34 ROIs), before and after addition of 200 µM NMDA. The results are expressed as Mean ± standard error. *: *p* < 0.05 compared to vehicle.

The regulation of intracellular [Ca^2+^] has an important role in the mechanisms underlying memory processes and synaptic dysfunctions [38]. Accordingly, we evaluated the effects of ASX on cytoplasmic [Ca^2+^] levels in cultured hippocampal neurons treated with NMDA. For this purpose, hippocampal neurons were transfected with the genetically encoded Ca^2+^ sensor GCamp3, and subsequently treated for 24 h either with vehicle (Figure 5A) or with 10 µM ASX (Figure 5B) prior to NMDA addition. Representative images, showing the probe fluorescence increase induced by incubation with 200 µM NMDA, are illustrated in Figure 5A,B). Line-scan images generated under an image acquisition rate of 1-s are presented in Figure 5A1,B1. The fast increase in probe fluorescence induced by NMDA addition (Figure 5A,A1) was inhibited by pre-treatment with ASX for 24 h (Figure 5B,B1). Subsequent addition of the Ca^2+^ ionophore Ionomycin effectively triggered a robust fluorescence increase in both conditions (IONO; Figure 5A1,B1). The graph presented in Figure 5C shows a time-lapse experiment, where the traces represent the quantification of the fluorescence levels measured in the cytoplasm of transfected hippocampal neurons (*n* = 7 neurons; a minimum of 5 cytosolic ROIs were analyzed in each neuron) treated with 200 µM NMDA. Preincubation with ASX reduced the fluorescence increase by 40% and induced NMDA by 200 µM, which reflects the increase in cytoplasmic [Ca^2+^], induced by 200 µM NMDA (*n* = 5 neurons, at least six cytosolic ROIs for each neuron), compared to control neurons incubated only with NMDA.

## 3. Discussion

Here, we investigated the possible protective properties of the natural antioxidant agent ASX, using a cellular model of neurotoxicity induced by addition of excitotoxic concentrations of NMDA to the SH-SY5Y cell line or to primary hippocampal neurons. We found that long-term treatment (24 h) with ASX induced cellular metabolic adaptations that inhibited the increase in [Ca^2+^] and ROS levels induced by the subsequent application of a cytotoxic stimulus (200 µM NMDA), thus normalizing the aberrant crosstalk between Ca^2+^and ROS signaling that is characteristic of cytotoxic conditions. First, we used a chemical Ca^2+^-sensitive probe to show that NMDAR activation in SH-SY5Y cells was dose-dependent. The present results showing NMDAR-mediated [Ca^2+^] increases in SH-SY5Y cells are consistent with our previously published study, in which we demonstrated that the addition of NMDA to primary hippocampal cultures elicits persistent neuronal Ca^2+^ signals that require functional NMDA receptors [21]. Accordingly, we consider that SH-SY5Y cells represent a suitable neuronal model for the present studies.

As reported in other studies [39,40], we found that a significant cytoplasmic[Ca^2+^] increase, such as that evoked by a high concentration of NMDA (200 µM), leads to sizable mitochondrial depolarization and excessive mitochondrial ROS generation [41], which leads to excitotoxicity and cell death due to an impairment in mitochondrial function. However, an NMDA concentration (16 µM) closer to the physiological range also increased both [Ca^2+^] and mitochondrial superoxide levels but did not affect cell metabolic activity (Figure 2B; Supplementary Figure S2). These Ca^2+^ and ROS dynamics under a physiological context point towards the role played by these species as second messengers needed for normal neuronal processes, such as synaptic plasticity [18]. In addition, we showed in SH-SY5Y cells that ASX provided enough antioxidant protection against the toxicity induced by H_2_O_2_ and the increase in mitochondrial ROS induced by incubation with NMDA. These results support our previous conclusion [42] that ASX has powerful antioxidant capacity. Moreover, in this work we found that incubation with ASX (≤10 µM) for ≤24 h was nontoxic to primary hippocampal neurons, evaluated by the live/dead assay.

Although superoxide anion is one of the major ROS involved in the generation of mitochondrial oxidative stress [43], overactivation of NMDAR has been linked also to the generation of ROS mediated by Ca^2+^-dependent enzymes, such as phospholipase A2, nitric oxide synthase and xanthine oxidase, all of which contribute to unbalance the oxidative state of mitochondria and induce excitotoxicity [44]. In our cellular model, the neuroprotective mechanism of ASX against the excitotoxicity induced by a high concentration of NMDA (200 µM) presumably entails the mitigation of mitochondrial oxidative stress. This suggestion is consistent with a recent study, which reported increasing oxidative stress in SH-SY5Y cells treated with glutamate, which led to cell death, probably via an apoptotic mechanism [11].

Several studies have indicated that ASX reduces mitochondrial ROS generation in vitro, and shown its consequences [45]. For instance, SH-SY5Y cells treated with MPTP display dysfunctions similar to those observed in Parkinson’s disease [46]. These dysfunctions are due to the inhibition of mitochondrial complex I, leading to free radical generation and the collapse of mitochondrial membrane potential [46]. Interestingly, ASX prevents both the ROS and mitochondrial dysfunction induced by this neurotoxin [46]. In accord, another neurotoxin, 6-hydroxydopamine (6-OHDA), which when administered intracranially is widely used as an experimental model of Parkinson’s disease, produces massive destruction of nigrostriatal dopaminergic neurons and motor dysfunctions [47]. SH-SY5Y cells incubated with 6-OHDA exhibit a significant decrease in cell viability as well as aberrant ROS generation, whereas pretreating these cells with ASX neutralizes these effects [45].

A study with HeLa cells expressing a mitochondrial redox-sensitive green fluorescent protein (roGFP1) reported that cells pretreated with ASX exhibited a lower increase in cellular oxidative state following hydrogen peroxide addition compared to untreated cells [12]. Another study reported that ASX prevents the increase in hydrogen peroxide levels induced by the addition of amyloid beta-peptide to primary hippocampal cultures, transfected with the HyPer-Mito probe to detect mitochondrial hydrogen peroxide levels [42]. Both studies support the proposal that ASX acts by mitigating the oxidative stress generated mainly in the mitochondria.

Even though ASX exerts its antioxidant activity by absorbing the energy of excited singlet oxygen or other radical species via the polyene chain present in its chemical structure [48], its action mechanism extends beyond ROS neutralization. Consistent with this idea, ASX has a strong transcriptional impact. In a cellular model of Parkinson’s disease-SH-SY5Y cells treated with MPTP-ASX enhanced cellular function possibly by a mechanism involving the increased expression of antioxidant enzymes (SOD, catalase) and anti-apoptotic proteins (BcL2), as well as decreased expression of the pro-apoptotic protein Bax [46]. In this context, it has been described that ASX decreases the expression of inflammatory genes like IL-6 in BV-2 microglial cells, by inhibition of the nuclear translocation of the transcription factor NF-kB [49].

Increasing evidence indicates that there is substantial crosstalk between ROS and Ca^2+^ signaling in different cellular systems [21,50,51]. The involvement of Ca^2+^ release mediated by redox-sensitive channels in the generation of postsynaptic Ca^2+^ signals, synaptic plasticity, and memory processes has acquired increasing relevance in the last years [17,28,52,53]. Moreover, in Alzheimer’s disease mutants, RyR channels mediate the generation of aberrant CICR dynamics in spines and dendrites elicited by the activation of NMDAR-dependent transmission; hence, these channels have been involved in the process of progressive memory loss associated with this pathology [29].

A previous report showed that NMDA addition to cortical neurons induces the sequential activation of the enzymes neuronal Nitric Oxide synthase (nNOS) and type-2 NADPH oxidase (NOX2), leading to enhanced ROS production [54], which in turn may promote the activation of redox sensitive Ca^2+^ release and of Ca^2+^-dependent signaling pathways. In fact, we have reported recently that the cytoplasmic [Ca^2+^] increase caused by NMDAR activation promotes nNOS and NOX2 activities; the ensuing ROS generation together with the cytoplasmic [Ca^2+^] increase lead to activation of Ca^2+^ release mediated by the type-2 RyR isoform [28].

To conclude, the present results add to the neuroprotective effects of ASX in preventing oxidative stress, inflammation, and aberrant gene expression in both excitotoxicity and neurodegenerative disease cell models [2,3,4]. Coupled with its ability to cross the blood–brain barrier, these properties have made ASX a promising molecule to be used as a neuroprotective and anti-aging agent, exerting direct actions onto the brain. Of note, the protective effects of ASX have been recently demonstrated in a subarachnoid hemorrhage condition [55], as well as in some clinical trials in which ASX has been observed to provide cognitive improvement [56]. A recent discussion of the effects of ASX supplementation on oxidative stress in humans is presented elsewhere [57].

## 4. Materials and Methods

### 4.1. Materials

Minimal essential medium (MEM), F12 medium, fetal bovine serum (FBS), non-essential amino acids and antibiotic-antimycotic (100X), Trizol reagent, B27 supplement, Neurobasal medium, Lipofectamine 2000 Transfection Reagent and Alexa Fluor 488-conjugated goat anti-mouse IgG (H+L) antibody were purchased from Invitrogen (Carlsbad, CA, USA). Fluo-4 AM, MitoSOX, Mitotracker green, Hoechst 33,342 stain and 3-(4, 5-dimethylthiazolyl-2)-2, 5-diphenyltetrazolium bromide (MTT) were purchased from Molecular Probes (Chicago, IL, USA). NMDA and D(−)-2-Amino-5-phosphonopentanoic acid (APV) were obtained from Tocris (Bristol, UK). Monoclonal Glur1/Nr1 antibody was obtained from Neuromab Antibodies Inc., (Davis, CA, USA). ASX- rich pigment (Supplementary Figure S1) was extracted from Lithodes antarcticus (supplementary methods; BIOTEX S.A., Santiago, Chile). pcDNA3-Cyto-GCaMP3 (Plasmid #64853: named in the text also as Gcamp-actin), was acquired from Addgene (Teddington, UK).

### 4.2. SH-SY5Y Cell Cultures

SH-SY5Y is a thrice cloned subline of the neuroblastoma cell line SK-N-SH established in 1970 from a metastatic bone tumor. SH-SY5Y cells (ATCC^®^ CRL-2266^™^) were seeded on a 96-well plate at a density of 20,000 cells/well (for viability assay, calcium, and mitochondrial superoxide measurements) or 40,000 cells/well per 12 mm diameter glass cover (immunocytochemistry and colocalization experiments) and grown in MEM/F12 medium supplemented with 10% FBS, 1% non-essential amino acids and 1% Anti-Anti (100X) antibiotic-antimycotic. Cells were grown in a saturated humidity atmosphere containing 95% O_2_ and 5% CO_2_, for 3 days at 37 °C.

### 4.3. Primary Hippocampal Cultures

Cultures were prepared from rat embryos at embryonic day 18 (E18) using a previously standardized protocol [21]. Briefly, the hippocampi were extracted in the sterile zone and maintained at 4 °C in Ca^2+^/Mg^2+^free Hanks saline solution containing in mM (135 NaCl, 5.4 KCl, 0.5 NaH_2_PO_4_, 0.33 Na_2_HPO_4_, and 5.5 D-glucose) balanced at pH 7.4. Subsequently, the hippocampi were trypsinized, washed with Hanks solution and mechanically dissociated in MEM 10 (MEM, 19.4 mM D-glucose, 26 mM NaHCO_3_, supplemented with 10% horse serum, 10 U/mL penicillin, 10 μg/mL of streptomycin). The non-disintegrated tissue fragments were sedimented by centrifugation at 800 rpm for 10 s; cells in suspension were recovered and seeded at a density of 40,000 cells per 12 mm diameter glass cover previously treated with poly-Lysine (50 μg/mL). MEM 10-cultured cells were kept at 37 °C in a humid atmosphere with 5% CO_2_; 60 min after, the medium was replaced by Neurobasal^®^ supplemented with B27, 20 U/mL penicillin, 20 μg/mL streptomycin and 2 mM Glutamax and were maintained under these conditions for 7 days. All experiments with rats were performed under established condition for FAWC (Farm Animal Welfare Committee); known as five liberties, under de guidelines ARRIVE of the National center for replacement, refinement, and reduction of experimentation animals (3Rs) of England, which is used by the Bioethics for Research and Animal Care institutional committee from Universidad de Valparaíso (CICUAL-UV).

### 4.4. Pharmacological Stimulation of NMDAR

To stimulate NMDAR, SH-SY5Y cells were seeded on a 96-well plate and grown for 3 days. After this period, cells were grown for 24 h in medium with 1% serum and then, cells were incubated with 16 µM NMDA (low concentration) or 200 µM NMDA (high concentration) in magnesium-free buffer (NMDA buffer) containing (in mM): 4.8 KCl, 118 NaCl, 3 CaCl_2_, 10 Glucose, 0.01 Glycine, 20 Hepes/Tris, pH 7.4. To inhibit NMDAR, cells were pre-incubated with 200 µM APV, an NMDAR antagonist, for 1 h before NMDA addition.

### 4.5. Immunocytochemistry

After culturing in normal medium for three days and one day with medium containing 1% serum, SH-SY5Y cells were washed twice with PBS and fixed for 20 min in PBS containing 4% paraformaldehyde and 4% sucrose. Then, cells were permeabilized for 10 min with 0.1% Triton X-100 in PBS. Cells were stained for NMDAR using anti-Glur1/Nr1 monoclonal antibodies (dilution 1:50). Following incubation with the primary antibody, cells were washed and incubated with Alexa Fluor 488-conjugated goat anti-mouse antibody (1:200). Nuclei were counterstained for 10 min with 2 μg/mL Hoechst stain. Cells were imaged with a Nikon Eclipse C180i laser scanning fluorescence microscope, using a multi-track configuration with two laser excitation lines and the following respective emission filters: 488 nm (515/30), for Alexa Fluor 488 and 350 nm (460) for Hoechst.

### 4.6. Cell Metabolic Activity Assay

Cellular metabolic activity was determined by the MTT colorimetric assay [36]. For experiments with NMDA, cells were cultured in 96-well plates and grown for 3 days. After this time, cells were grown in medium with 1% serum in the presence of 10 µM ASX or vehicle (absolute ethanol 2% *v/v*) for 24 h. Cells were rinsed and incubated with 16 µM NMDA or 200 µM NMDA during 0, 30, 60, 90 or 120 min in NMDA buffer. Subsequently, the cells were washed and kept in medium with 1% serum for 24 h after treatment with NMDA. Cells were incubated with MTT (5 mg/mL) in culture medium with 1% FBS for 4 h; 0.01 M SDS-HCl was added to dissolve MTT crystals. Finally, absorbance was measured with a multimode microplate reader Synergy, HT at the wavelength of 570 nm. For experiments with hydrogen peroxide, the same protocol described above was performed, except that cells were treated for 1 h with 50 µM H_2_O_2_ instead of NMDA in medium with 1% serum.

### 4.7. Intracellular Ca^2+^ Measurements

To detect changes in intracellular [Ca^2+^] elicited by NMDAR activation, SH-SY5Y cells were incubated with 1.5 µM Fluo-4 AM for 30 min at 37 °C in extracellular magnesium-free buffer that contained in mM: 4.8 KCl, 118 NaCl, 3 CaCl_2_, 10 Glucose, 0.01 Glycine, 20 Hepes/Tris, pH 7.4. The cells were washed with an extracellular buffer for NMDA experiments to remove probe excess. The basal fluorescence intensity was continuously monitored (λ Ex/Em: 496/506 nm) during 4 min before and 20 min after NMDA addition, using the multimode microplate reader Synergy, HT (Biotek). Fluo-4 fluorescence levels were expressed as ∆F/F_0_, where F_0_ corresponds to the average basal fluorescence acquired during 4 min prior to NMDA addition, while ΔF corresponds to the difference between each fluorescence value measured after NMDA incubation (F) and F_0_.

### 4.8. Mitochondrial ROS Measurements

To determine mitochondrial ROS levels following NMDAR activation cells were incubated with 5 µM MitoSOX for 30 min at 37 °C. This Live-cell permeant fluorogenic superoxide-sensitive dye targeted the mitochondria selectively and rapidly. The fluorescence intensity before and during the experiment was measured at λ Ex/Em: 510/580 nm. The colocalization experiments were performed in the confocal microscope (Nikon C1 Plus) to evaluate the correct location of MitoSOX by comparison with the subcellular mitochondrial marker Mitotracker green and with Hoechst as the nuclear counterstain. The incubation and preparation of the dyes was performed according to manufacturer instructions. The effects of ASX on mitochondrial ROS levels were assayed in SH-SY5Y cells pre-incubated with increasing concentrations of ASX (in µM: 0.0005; 0.001; 0.01; 0.1; 1, 10) in culture medium supplemented with 1% serum for 24 h before NMDA addition. Treatment with NMDA was performed at the time of the experiment; NMDA was added to the extracellular medium as described for the calcium measurements.

### 4.9. Determination of Cytoplasmic Ca^2+^ Signals in Primary Hippocampal Neurons

Primary hippocampal neurons (7 days-in-vitro, DIV) transfected with the GCamp-actin EGFP vector were treated by 24 h with 10 μM ASX. Next, control cells (without treatment) and ASX-treated cells were transferred to extracellular solution for NMDA experiments (in mM: 4.8 KCl, 118 NaCl, 3 CaCl_2_, 10 Glucose, 0.01 Glycine, 20 Hepes/Tris, pH 7.4) and were treated with 200 μM NMDA. Fluorescence images corresponding to intracellular Ca^2+^ signals were obtained every 5 s using a confocal microscope (Nikon C2; Melville, NY, USA) and were processed using the NIHWCIF Image J (National Institutes of Health) software. Image data were acquired from different regions of optical interest (ROI) located in cell bodies and dendrites. Frame scans were averaged using the equipment data acquisition program. Fluorescent signals were expressed as ΔF/F_0_ values, where F_0_ corresponds to the basal fluorescence before the addition of any treatment. The increase in probe fluorescence (intracellular [Ca^2+^]) induced by 200 μM NMDA did not saturate the signal of the sensor. All experiments were done at room temperature (20–22 °C).

### 4.10. Statistical Analysis

Data are expressed as means ± standard error. One-way ANOVA or Bonferroni’s Multiple Comparisons test were used for data analysis. Differences were considered significant at *p* < 0.05.

The results were analyzed using a reading test provided by the GraphPad Prism 5 software. Statistical significance was considered at *p* < 0.05.

## Supplementary Materials

The following are available online at https://www.mdpi.com/1660-3397/18/6/335/s1, Figure S1: High performance liquid chromatography (HPLC) of a free trans astaxanthin, Figure S2: Cell viability assay of primary hippocampal cultures incubated with NMDA and/or ASX, Figure S3: Representative immunofluorescence images showing the negative control for anti-NMDAR subunit GluR1/N1 antibody) and supplementary methods.
