# Supplementary material for: Astaxanthin Counteracts Excitotoxicity and Reduces the Ensuing Increases in Calcium Levels and Mitochondrial Reactive Oxygen Species Generation
Source: Mar Drugs. 2020 Jun 26;18(6):335. doi: 10.3390/md18060335 (PMC7345213; doi:10.3390/md18060335)
Supplement: Supplementary file 1 [file marinedrugs-18-00335-s001.pdf]

SUPPLEMENTARY FIGURES

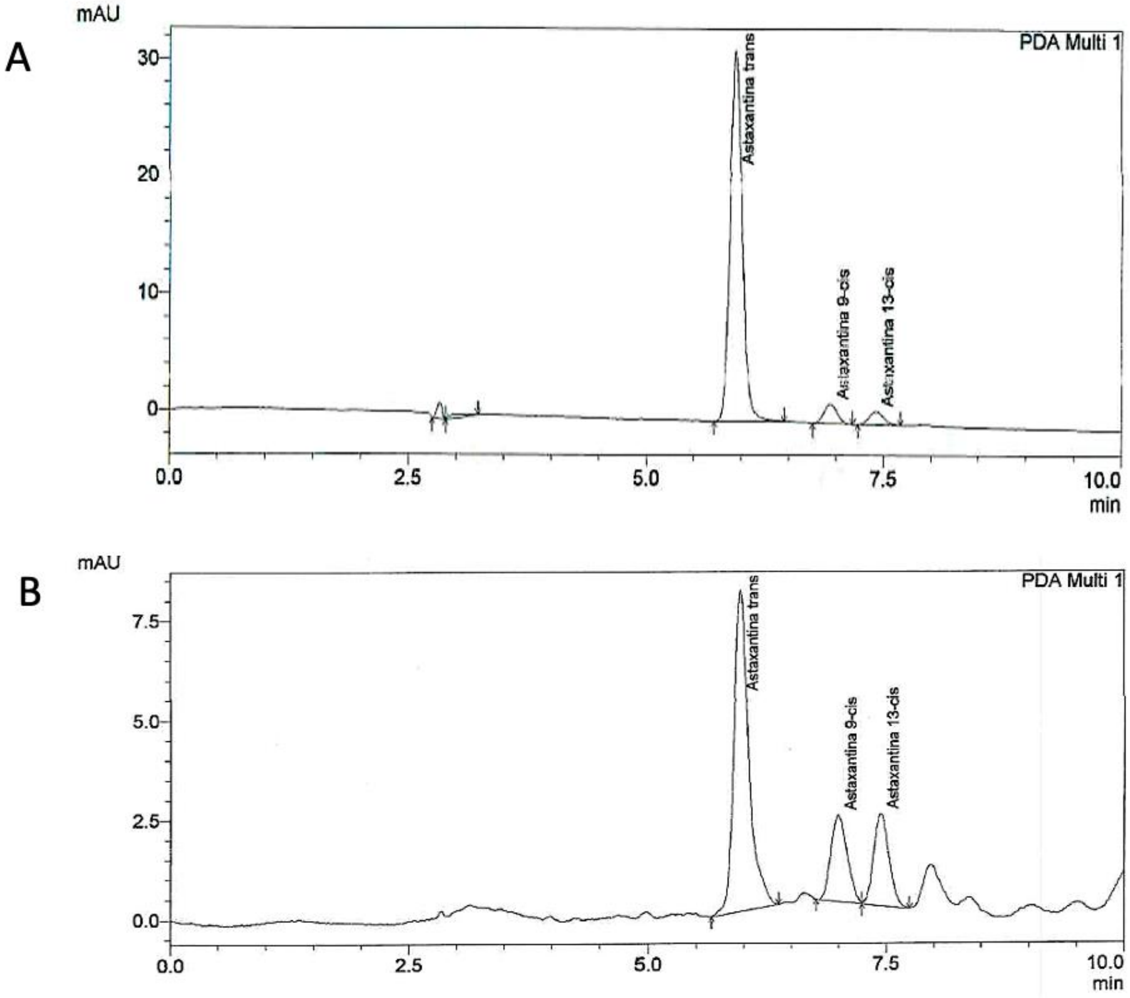

Supplementary Figure 1. A) High performance liquid chromatography (HPLC) of a free trans astaxanthin standard at 5.9 min retention: the chromatograph also shows 9-cis astaxanthin and 13-cis astaxanthin, retention times of 6.9 and 7.4 min, respectively, which are the isomers of this natural pigment. B) HPLC of a sample of free astaxanthin obtained from the pigment-rich extract used in this work, which displays a retention time of 6.0 min: the 9-cis and 13-cis isomers have retention times at 7.0 and 7.4 min.

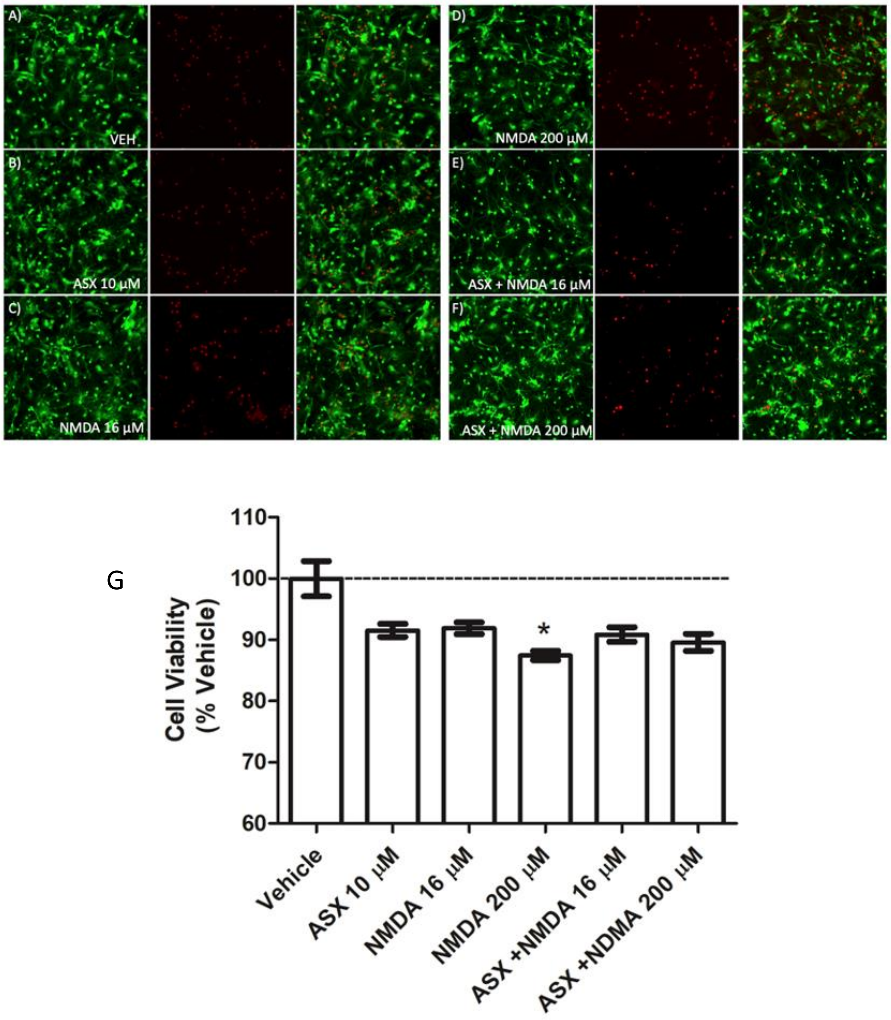

Supplementary Figure 2. Cell viability assay of primary hippocampal cultures incubated with NMDA and/or ASX. Representative images of 13-15 DIV hippocampal primary cultures obtained after performing the Live / Dead® cell viability assay. Cells were incubated for 24 h in the presence of vehicle (A) or were treated for 24 h with 10 µM ASX (B), followed by addition of 16 µM NMDA for 5 min (C), 200 µm NMDA for 5 min (D), or were preincubated with 10 µM ASX and subsequently treated with 16 µM or 200 µM NMDA for 5 min, respectively (E and F). The live cells were identified by Calcein-associated green fluorescence and the dead cells were stained by ethidium-associated red fluorescence, linked to DNA. Scale bar: 50 µm. (G) The graph shows the quantitative analysis of cell viability, results are shown as percentages of cell viability relative to the viability of the vehicle-treated culture (experimental control). Graphed values correspond to the mean ± SEM obtained from three independent experiments (each condition performed in triplicate), with different neuronal cultures. Statistical analysis was performed by One-way ANOVA, followed by Dunn post-test to determine statistically significant differences (* $p < 0.05$ compared to the control).

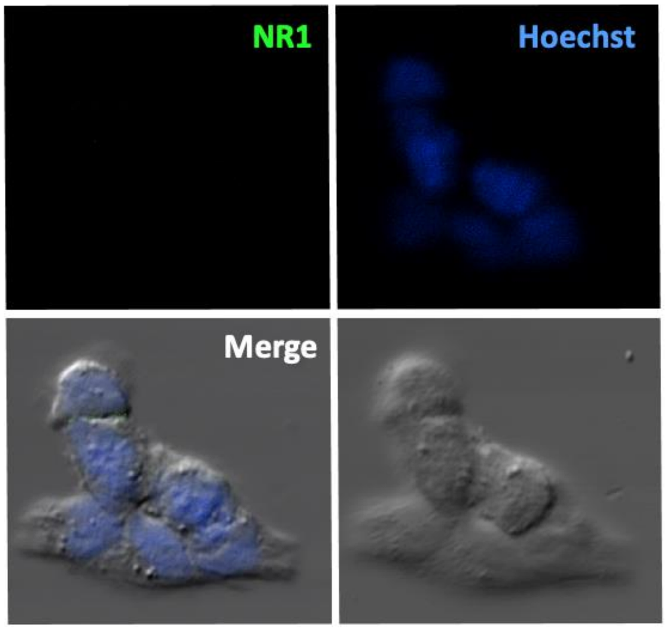

Supplementary figure 3. (A) Representative immunofluorescence images showing the negative control for anti-NMDAR subunit GluR1/N1 antibody. Cells were fixed, permeabilized and incubated only with the secondary antibody (green). The nucleus was stained with Hoescht (blue).

SUPPLEMENTARY METHODS

Astaxanthin-rich pigment extraction method: The method consisted of an extraction from spider crab shell, with n-hexane and ethanol at a ratio of 1: 5. The mixture was stirred with a stainless-steel stirrer at a speed of 60 rpm for 5 h. The liquid phase was filtered, and the solvent was evaporated by spray drying protected from light. The lipid fraction, rich in pigments, was concentrated on a rotavapor under a nitrogen environment at 30 °C in a methanol-acetonitrile mixture (7.5: 92.5 v), in the absence of light. The absorption spectrum of the astaxanthin derivatives, obtained in a visible ultraviolet spectrophotometer, showed a typical peak at 473 nm in 14% acetone in hexane and at 486 nm in chloroform. The chromatograms were obtained on a Waters TM600-E HPLC, with a diode array detector, controlled by the Millennium 2010 software.

Cell Viability Live/Death Assay: Neuronal hippocampal cultures (13–15 DIV) were incubated with 10 μM ASX or vehicle. Twenty-four hours later, cells were treated with 16 μM or 200 μM NMDA for 5 minutes in the presence of ASX or Vehicle. After, NMDA was washed, and cells were maintained for 24 h. Cell viability was evaluated with the live/dead kit following the manufacturer's instructions, as previously described. Briefly, after removal of the culture medium cells were gently washed three times with warm PBS-glucose and incubated at room temperature

for 30 min in the presence of 2 μM Calcein-AM ester and 1 μM ethidium homodimer in PBS-glucose. Live neurons were identified by green Calcein fluorescence, and dead neurons were identified by the red fluorescence of DNA-bound ethidium. Cells were examined on a Nikon® Eclipse Ti-Eat at 20x magnification. The images were analyzed, and cells were counted with the cell counter plugin of the Image J software, thus the green or red stained cells were counted with the plugin cell counter. At least three random fields were imaged per culture well (three replicate wells were used per experimental condition in each experiment), and about 300 cells were counted in each well. Three independent tests were performed with different neuronal cultures. Cell viability was expressed as the percentage relative to the untreated vehicle (control) cultures.
